# Lay perceptions, beliefs and practices linked to the persistence of anthrax outbreaks in cattle in the Western Province of Zambia

**DOI:** 10.4102/ojvr.v85i1.1615

**Published:** 2018-08-29

**Authors:** Doreen C. Sitali, Mwamba C. Twambo, Mumba Chisoni, Muma J. Bwalya, Musso Munyeme

**Affiliations:** 1 Department of Disease Control, University of Zambia, Zambia; 2 Department of Veterinary Services, Ministry of Fisheries and Livestock, Zambia

## Abstract

Anthrax, a neglected zoonotic disease that is transmitted by a spore-forming, rod-shaped bacterium, *Bacillus anthracis*, has reached endemic proportions in the Western Province of Zambia. Transmission of anthrax from the environment as well as between cattle has been observed to be partly because of entrenched beliefs, perceptions and traditional practices among cattle farmers in the known outbreak areas. This study was aimed at exploring lay perceptions, beliefs and practices that influence anthrax transmission in cattle of the Western Province. A mixed-methods study was conducted from August to December 2015. Quantitative data were collected using a cross-sectional survey. Qualitative data were generated by interviewing professional staff and community members. Five focus group discussions and five key informant interviews were conducted. Thematic analysis of interview data was performed using NVivo software. The findings suggested that cattle anthrax was biologically as well as culturally maintained. Cattle farmers were reluctant to have their livestock vaccinated against anthrax because of perceived low efficacy of the vaccine. Also, the cattle farmers did not trust professional staff and their technical interventions. Popular cultural practices that involved exchange of animals between herds contributed to uncontrolled cattle movements between herds and subsequent transmission of anthrax. These findings imply the need for professional staff to be culturally competent in handling socio-cultural issues that are known to be barriers for disease control in animals. There is a need to develop a policy framework that will foster integrated control of anthrax across disciplines.

## Introduction

Anthrax is a severe infectious disease caused by a bacterium known as *Bacillus anthracis*. Anthrax can be found naturally in soil and commonly affects domesticated and wild herbivores. Although anthrax is perceived to be a weapon of bioterrorism in most countries in the world (Siamudaala et al. 2006), it has ecologically emerged to be a significant public health threat in the Western Province of Zambia. In this region, the disease has reached endemic proportions with interminable outbreaks in both cattle and humans (Siziya 2017). Between 1989 and 1995, the Western Province recorded 1626 suspected cases of cattle anthrax of which 51 were confirmed cases (Siamudaala et al. 2006), and 1216 cases were recorded between 1999 and 2007 (Munang’andu et al. 2012). Furthermore, Zambia is categorised under the countries where the problem of anthrax in Africa has reached a hyper-endemic state given the incessant and the long-drawn-out outbreak periods that seem unceasing in the recent years (World Anthrax Data Site 2007). The disease has had a modifying effect across families, public health institutions and ecotourism in the affected areas of the Western Province as well as other parts of Zambia, such as the Luangwa Valley (Kamboyi 2015). Despite the Western Province being the most sparsely populated place in Zambia, with a human population density of less than five people per square kilometre (IUCN 2003), outbreaks and occurrence of anthrax in cattle are markedly more noticeable in this area than any other area in Zambia. The Western Province has recorded six outbreaks of anthrax in cattle and humans between 2011 and 2016 compared to Southern Province that has not recorded any outbreaks since 2011 (Office of the Auditor-General 2015). The cattle population in the Western Province is estimated to be over 760 000 but is continuously under serious threat not only to anthrax but also to contagious bovine pleuralpneumonia among other diseases (National Livestock Epidemiology and Information Centre 2015). The place has the highest incidence and prevalence of anthrax in Zambia, with cultural practices and beliefs of the local people being identified as significant risk factors coupled with the ecological set-up (Kamboyi 2015; Munang’andu et al. 2012; Siamudaala et al. 2006). The disease endemicity is a result of appropriate mix of environmental and epidemiological factors. Ecological factors include the cyclical rainfall pattern, flooding, evaporation potential, temperature and the geology of the floodplains. Epidemiological factors include the increase in cattle and human populations on the floodplains during the dry season, leading to anthropogenic pressure, transhumance grazing system (Munang’andu et al. 2012) and human behavioural factors (Sitali et al. 2017). Notwithstanding that ecological and epidemiological factors have been researched to some extent, human practices and behavioural factors have not been scrutinised and are still poorly understood (Mumba et al. 2018). Outbreak investigation reports and reviews of anthrax outbreaks in the Western Province have indicated that the disease has persisted in the province because of entrenched cultural beliefs and practices of local communities (Mwambi et al. 2017). However, this phenomenon has been poorly explored thus far. It is crucial for programme implementers to consider local beliefs, practices and perceptions surrounding the disease if control measures are to yield the most significant results. Modifying factors such as demographics and socio-economic status, among others, are vital in predicting how a community responds to information leading to health behaviour. In this study, the overall objective was to assess and determine the perceptions, beliefs and cultural practices, and other anthropogenic factors, related to the contraction of anthrax by cattle in the Western Province. These factors have been partly discussed in a previous paper (Sitali et al. 2017); in this article, we describe the observational and situational analysis data of anthrax in the Western Province as it relates to the endemicity in cattle, zoonotic implication and public health impact of the disease.

## Materials and methods

### Study area

**FIGURE 1:**
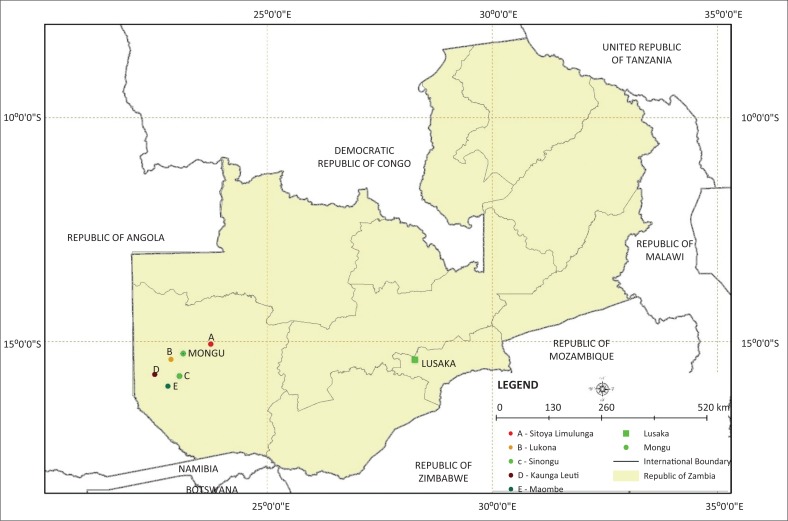
Map of Zambia showing four districts of the Western Province.

Three districts that are endemic to anthrax in the Western Province were conveniently selected for the study. These were Mongu, Nalolo and Limulunga (Figure 1). The Western Province lies in the upper Zambezi basin also called the Barotse floodplain located at coordinates 14°19’–16°32’S and 23°15’–23°33’E and covering about 5500 km^2^ in extent (IUCN 2003). The maximum flooded area is estimated at 10 750 km^2^ (Welcomme 1975) when floods of all tributaries of the Zambezi River are taken into account. The floodplain stretches from the confluence of the Lungwebungu River with the Zambezi River in the north extending southwards for a distance of 250 km until Ngonye falls. Soils are composed of the Kalahari sands stretching several metres deep underlain by calcareous rocks (Munang’andu et al. 2012). Elsewhere, calcareous soils have been associated with prolonged survival of anthrax spores (Hugh-Jones & Hussaini 1975; New et al. 2002). The main human activities in the study area are traditional cattle farming, fishing and rice farming, and to a lesser extent, maize and cassava farming in the upper forest lands. The cattle population in the study area is estimated at over 760 000 (National Livestock Epidemiology and information Centre 2015). The Zambezi floodplain alone contains a population of over 225 000 people in an estimated 28 000 sparsely spaced households (Central Statistical Office Zambia 2014). Given the landmass of the floodplain to be 10 750 km^2^ at its maximum (Welcomme 1975), this gives a human population density of 20.6 people/km^2^ in the floodplain during the dry season. However, it was estimated that another 200 000 people live on the plain margin (Turpie 2004) and their livelihood is also dependent on the floodplain, thereby exerting more pressure on the natural resource utilisation of the floodplain.

### Study design

The study was a mixed-methods design employing both quantitative and qualitative methods. A cross-sectional survey was concurrently conducted with five focus group discussions and five key informant interviews from August to December 2015. Quantitative data were reported in a paper by Sitali et al. (2017).

### Data collection and analysis

Respondents for the focus group discussions were purposively sampled from the survey participants based on whether they had lost cattle because of anthrax, had a family member who suffered from anthrax or died from the same. Five key informant interviews with professional staff were held as follows: one with a senior veterinary officer, three with veterinary assistants and one officer working for the Ministry of Health. An unstructured topic guide with open-ended questions was used to guide the interviews. The interview guide focused on the experiences of key informants with communities in controlling for anthrax, and the challenges faced. All the interviews took place in the respondents’ environments, either their office or home. The interviews lasted for a minimum of 30 min to 1 h. Also, five focus group discussions were held with community members as follows: one in Limulunga, two in Mongu and two in Nalolo District. Each focus group had an average of 11 participants. Two of the focus group discussions were held with female participants only; one was held with men only to identify gender differences in perceptions and practices. Two of the focus group discussions were held with both sexes. Field observations were supplemented with informal discussions with community members and professional staff from the health and livestock departments. A topic guide was used to guide the discussions, focusing on the beliefs, and perceptions, of respondents towards professional staff and control measures. Common cattle-rearing practices and cultural practices surrounding anthrax were also explored. Piloting of the tools was not performed as is the standard practice in qualitative research. However, at the end of each field day, recorded narratives were audio played to identify areas that needed further probing or clarification. The questions in the interview guide were then adjusted accordingly. This was repeated until saturation of data (no new themes emerge from data) was reached.

Interviews were audio-recorded. All the interviews were moderated by the principal investigator and a research assistant took field notes. Focus group discussions were either held at a village or crush-pen. Focus group discussions were held in the local language (*Lozi*) to facilitate understanding. The narratives were audio-taped and later translated into English and transcribed into computer files. Nvivo 12 for windows was used to help manage the data. Broad coding followed by fine coding was performed in NVivo software to facilitate the identification of themes. Framework matrices were formulated to help cross-check information from community members with that of professional staff to identify similar and contradicting views between professional and community members. Illustrative quotations that represented the themes were used to present results. Information was summarised through a logical risk chart of pathogen transmission and contamination based on the lay practices, beliefs and cultural understanding of the disease.

### Ethical consideration

The study received ethical approval from the University of Zambia Biomedical Research Ethics Committee (UNZABREC), protocol number 013-08-15. Permission to collect data was obtained from the Provincial Veterinary Officer of Western Province. Informed verbal consent was obtained from all the participants before interviewing them.

## Results

The results presented in this article were from the focus group discussions, key informant interviews, field observations as well as review of annual veterinary records. Four major themes and three sub-themes were generated from the focus group discussions and interviews. These were:

- popular beliefs of community members
- perceptions of community members
- common practices of community members
  ■ cattle-rearing practices
  ■ carcass disposal practices
  ■ cattle vaccination practices
- factors influencing beliefs and practices.

### Popular beliefs of community members

Farmers believed that the vaccine made their animals sick instead of protecting them from anthrax. According to them, cattle continued to die even after being vaccinated. Therefore, community members did not consider the vaccine to be effective in protecting their animals. One female respondent had this to say about cattle vaccination:

‘We have observed that the vaccines are injected in our cattle but what we have seen is that the disease continue to escalate. Because just after vaccination the cattle would die and again a week after, another.’ (FGD 2, male farmer, aged 52)

A senior veterinary staff explained that this belief was as a result of the manner in which government conducted anthrax vaccinations. He explained that anthrax was classified as a management disease which meant that the farmer bore the cost of vaccination as opposed to diseases of National Economic Importance (DNEIC) such as contagious bovine pleuropneumonia (CBPP) for which the government sponsors annual vaccination. However, most cattle farmers preferred to be reactive rather than proactive because they were only willing to have their cattle vaccinated against livestock diseases once there were cattle mortalities. Because there were usually no clinical signs observed before the cattle died, most animals at risk may already have been infected such that once vaccinated, some of those already infected succumbed to the disease as the vaccine itself is a live attenuated strain.

Also, community members believed that veterinarians introduced anthrax into their animals when they went to vaccinate the animals. One key informant explained how this belief came about in the area where he was working. He said:

‘…They have various reasons to give; their belief is that, when veterinary people come to their area to vaccinate animals, they bring diseases. Our animals usually die after they have vaccinated them. Moreover, this belief, it is like it emanated from the early years of 1970s when the first outbreak of CBPP occurred in Western Province. Because the vaccine that was being used then, the route of injection was the tail swish, so animals reacted by losing their tails, then because of that they believed that veterinary staff brings diseases to their animals.’ (Key informant 5, male, Veterinary Officer, aged 38)

**FIGURE 2:**
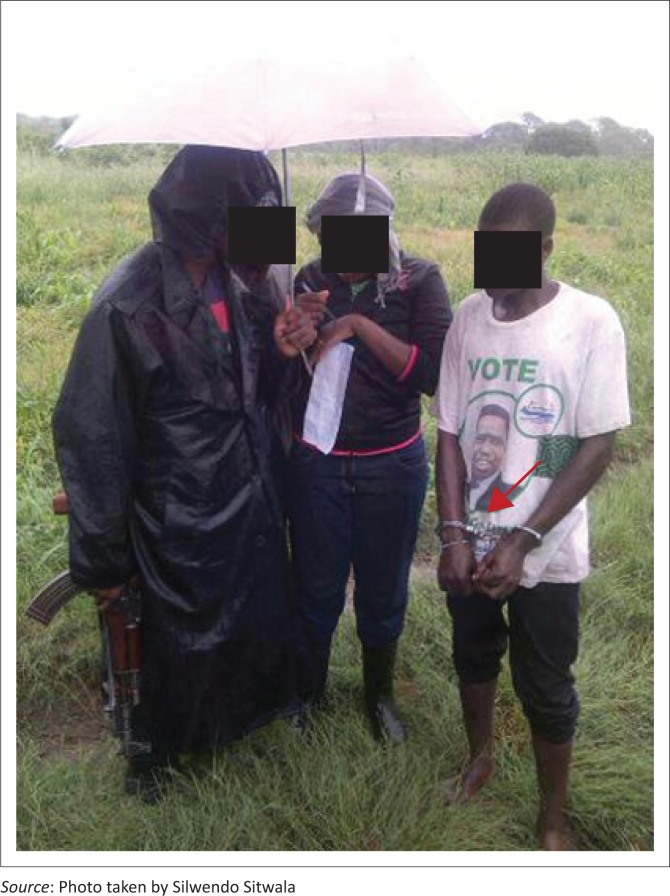
Cattle owner handcuffed by police officer to allow for cattle vaccination.

Because of these beliefs, most farmers avoided taking their cattle for vaccinations because of fear of losing them from anthrax. Some key informants explained that the situation in some veterinary camps was so severe that the veterinary officers had to work with police officials during vaccination campaigns. The police officers had to handcuff some of the resisting farmers to get their animals vaccinated. Sometimes, police officers were required to protect veterinary officers against hostile farmers (Figure 2).

### Perceptions concerning veterinary staff

From the focus group discussions, it was also evident that community members had certain perceptions concerning veterinary staff. Respondents suspected that veterinary staff worked with a reputed meat-processing company to wipe out their cattle population from their province. Veterinary staff were also suspected to be practicing *Satanism* (*Satanism* is a group of ideological and philosophical beliefs based on Satan). This suspicion came as a result of veterinary staff collecting blood samples from animals for sero-surveillance for CBPP. One key informant explained what commonly happened in his veterinary camp, he said:

‘The other part again is where you do sero-surveillance, when you are doing sero-surveillance; you have to collect blood samples…… So those when you collect blood from the animals they say these vets are practicing Satanism, where do they take the blood of our animals? So again even then, we had to work with the police to convince them to take blood samples. Usually, after you have handcuffed someone, usually they give in.’ (Key informant 5, male, Veterinary Officer, aged 38)

### Common practices among community members

#### Cattle-rearing practices

Cattle are the primary source of livelihood for most families in the Western Province. Cattle are sold to pay for school and medical fees, household necessities and dowry (*lobola*), and are used for draught power and manuring crop fields. Because of this, cattle loaning and exchange is common. Most farmers in the province also sell their cattle to a meat-processing company that is based in the provincial capital. It is therefore common for most farmers to trek their cattle on foot to the central abattoir. During trekking, farmers commonly use bush tracks that lead to interchange of diseases among cattle herds they directly encounter on the way or share grazing areas with. Also, when *lobola* is paid in the form of cattle, these have to be trekked to the woman’s family. Sometimes, cattle are loaned to another herder to take care of under the *mafisa* agreement (see below) and therefore had to be taken to the plains for manuring crop fields and to plough the fields. These scenarios lead to uncontrolled animal movements that result in the spread of animal diseases including anthrax. In the period before 1991, government had workers who manned and controlled animal movements and had infrastructure to regulate movement of cattle. These were laid off during the structural adjustment programme that was undertaken in 1991 (Muuka et al. 2012). This led to vandalism of the infrastructure and breakdown in animal movement controls. One participant explained the scenario in his village and said:

‘The problem of anthrax we do not have a law on the movement of cattle. So the cattle that bring the diseases are those from Shang’ombo (a neighbouring district). In the past there used to be buffer zones from Shang’ombo there, there used to be camps, so those people used to control the spread of diseases, but today the fences (cordon lines) have been damaged.’ (FGD 3; male participant; farmer; aged 62)

In the Western Province of Zambia, cattle farmers commonly practice a traditional cattle risk-management system called *mafisa.* In this system, farmers apportion out their cattle to other people to herd as a way of reducing the risk of losing animals from diseases and cattle raiders (Sumbwa 2000). If the person herding the cattle under the *mafisa* agreement takes good care of the animals, and they multiply, they are rewarded with an animal at the end of the year. Therefore, the system also served as a form of social support. However, if a cattle has died while under the care of a herder, he was obliged to produce evidence that it was not sold but died on its own. The herder is expected to dry the meat and or preserve the skin (Figure 4 left) and horns for the owner’s inspection to prove that he did not sell the animal. This practice has led to persistence of anthrax spores in the environment as the spores have been found to survive in these animal products for years. One respondent in a focus group discussion explained what happened in the *mafisa* agreement in *Bulozi.* He said:

‘It can die (meaning cattle) at the mafisa the one keeping it will not do anything about it until the owner comes. I can say that sometimes the owner of the cattle for mafisa is not around, went somewhere, because of fear of getting into trouble what will he do, just skin that cattle and dry it to wait for the owner to come and get it when he is back.’ (FGD 3 participant, male, farmer, aged 56)

In some instances, calves that are born within the *mafisa* contracts are taken back to where the owner lived. In this way, infected calves transmitted anthrax from one herd to the other. It is also a common practice for some farmers to send their animals to the plains for manuring crop fields and therefore cattle diseases were also transmitted between herds. Furthermore, sometimes, farmers distribute their animals to other relatives or areas when they heard rumours of a disease. If the cattle were already infected, they transmitted the disease to other herds.

This study also observed that uncontrolled cattle movement was a common practice in the province. Cattle were commonly used to pay *lobola.* Therefore, once a woman was married off, cattle were moved to the woman’s family. Moreover, cattle were also commonly used as a form of payment to traditional healers when one fell ill. Because of these trade-offs, cattle were commonly moved between herds, leading to possible transmission of animal diseases including anthrax.

#### Carcass disposal practices

Farmers also explained that because veterinary officers rarely responded to reports of animal mortalities, they attempted to diagnose the cause of death in cattle themselves. Therefore, it was common for farmers to open a carcass and examine for an enlarged spleen in the animal. This is usually performed in the plains where animals were being grazed. This practice therefore leads to exposure of the vegetative cells into open air (oxygen) and forms spores which contaminate the grazing areas and water points ready for infecting another animal. One respondent had this to say about carcass disposal:

‘What I want to say is that us that have cattle the problem we face is caused by the vet, we do not have a vet officer here nearby to diagnose the cause of death of the cattle and what disease it was suffering from so that we know what to do. So when it dies, we just get it and eat because we do not know what sickness it had.’ (FGD 1 participant, female, aged 47)

**FIGURE 3:**
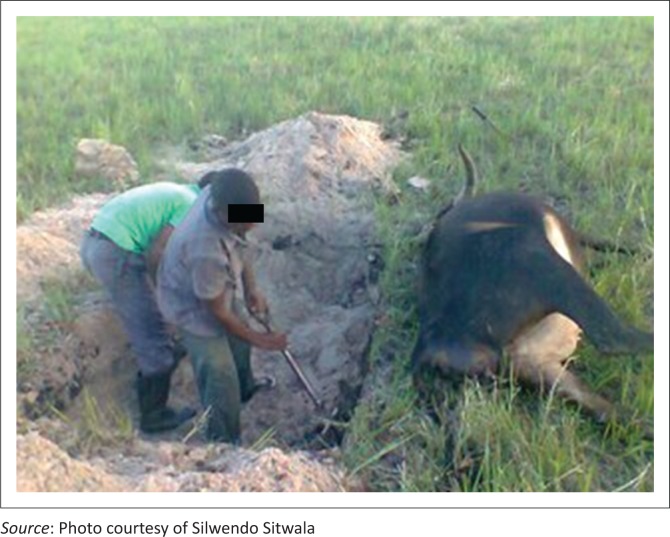
Community members burying anthrax carcass in grazing field.

**FIGURE 4:**
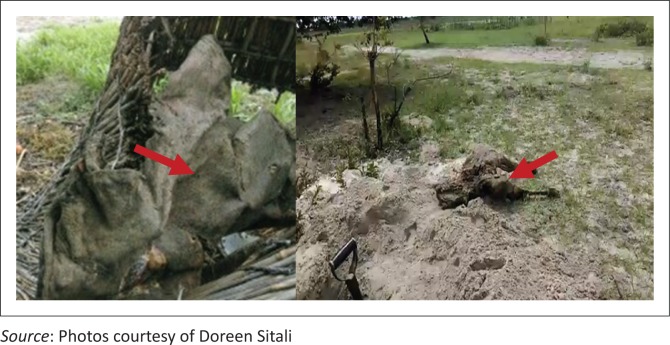
Preserved hide from anthrax carcass (left); exhumed remains of incinerated anthrax carcass (right).

Commonly, most farmers eat contaminated meat and dispose of cattle’s remains by burying (Chanda, Mulubwe & Mwale 2017; Chavwanga 2013). Disinfection of burial sites or carcasses is not performed, facilitating disease transmission. This was because, most of the times, veterinary staff are not available to supervise carcass disposal because of logistical challenges of transport and lack of fuel for incineration. Villagers butcher the animals and share the meat among themselves because of poor access to veterinary services. This practice leads to the dissemination of anthrax bacterium and contaminates the environment where other cattle are grazing (Figure 3). In instances where veterinary staff supervised disposal of the carcasses, the common method of disposal is by burying without decontamination of sites. This is because of lack of fuel for incineration and scarcity of firewood in the floodplains, and lack of disinfectants such as formaldehyde. Often, community members exhume cattle remains (Figure 4 right) and consume them because of high poverty levels. Incineration is the recommended method of choice disposal of anthrax carcasses (Food and Agriculture Organisation of the United Nations 2003).

#### Cultural practices affecting cattle-rearing

In the Western province, the local inhabitants depend on a transhumance grazing system that is tied to the flooding regime. Annually, herdsmen along with their families take cattle into the wetlands during the dry season (June–November). Therefore, at the peak of the dry season, cattle herds are concentrated around the lagoons and oxbow lakes. As the grass becomes overgrazed, cattle inhale the spores from the grass. They also ingest spores by drinking water from the lagoons where spores have been concentrated by incubator conditions (Munang’andu et al. 2012). This migration sometimes transmitted anthrax between the floodplains and the highland areas.

#### Cattle vaccination practices

The study also established that most farmers in the province did not vaccinate their cattle against anthrax. When provincial veterinary records for animal vaccinations were checked, it was found that only about 10% of the farmers vaccinated their cattle. Most key informants explained that most farmers avoided having their cattle vaccinated because of misconceptions and beliefs concerning the vaccine and veterinary staff. One veterinary camp officer explained how he experienced resistance from farmers in his district. He said:

‘What we realize in … [Name withheld] is that people are very difficult there … There is a lot of non-compliance by the farmers to have their animals vaccinated. Even despite this veterinary assistant acquiring some vaccine, others were still resisting to pay for the animals. They have various reasons to give; their belief is that, when vets come to our area to vaccinate the animals, they bring diseases. Our animals usually die after they have vaccinated them …’ (Key informant 1, Male, Veterinary officer, aged 62)

Apart from misconceptions and beliefs, most veterinary staff indicated that several logistical barriers affected farmers’ ability to have their cattle vaccinated. Among the most notable ones were the lack of cold chain equipment in the veterinary camps and low socio-economic status of farmers. One farmer said:

… the issue that affects us is that money is difficult to find with the poverty we have in our homes even just to manage to pay for one cattle to be vaccinated is a challenge … (FGD 2 participant, farmer, aged 77)

Other barriers were reduced access to the vaccine and lack of funding for logistical support for vaccinations and geographical remoteness of most areas. These barriers were further compounded by lack of government funding for anthrax control as a result of the disease being classified as a management disease. Also, communities had inadequate access to social services such as schools, health facilities and markets and therefore felt neglected. They lacked information on how anthrax affected their animals and how they got affected themselves. Because of this, most of them had poor health promotion–seeking behaviours such as not having cattle vaccinated.

In the Western Province, it was common for most cattle herds to be owned by several members of a kinship, some of whom lived away from the village or even overseas. Therefore, decisions to have cattle vaccinated were often delayed because they needed to be made collectively. Also, some veterinary staff explained that most farmers were of low educational status and did not understand the vaccination schedules. Therefore, because of poor understanding of the schedules, most farmers thought that their cattle were protected against anthrax once they received vaccinations for other diseases. Table 1 summarises the findings in a logical risk chart.

**TABLE 1:** Barriers identified to affect anthrax outbreaks in the Western Province of Zambia.

Domain	Veterinary staff	Anthrax vaccine	Cattle-rearing practices	Cattle disposal practices	Cattle vaccination practices
Perceptions	Veterinary staff are *Satanists*	Anthrax vaccine is not efficacious	nd	nd	-
Beliefs	Veterinary staff introduce anthrax into cattle Veterinary staff work with a reputed meat-processing company to wipe out the cattle population in the province	Vaccine makes cattle sick	Refractory to western advice	nd	Refractory to western advice
Practices	Wrong timing to intervene when farmer has lost an animal (need standard timing during extension services and education) Reliance on ‘traditional advisors’	nd	Traditional practices like *mafisa* Uncontrolled animal movements between areas Transhumance animal husbandry system	Unsupervised handling of carcasses Unsupervised disposal of carcasses Unsupervised handling of animal products	Unwilling to vaccinate cattle Lack of awareness of importance of vaccination

nd, not detected

## Discussion

This article set out to explore the lay beliefs, perceptions and practices linked to the persistent transmission of anthrax in cattle in the Western Province.

### Lay perceptions and beliefs

The study observed that community members had certain beliefs and perceptions that influenced their response to technical interventions. Firstly, community members had a negative attitude towards the anthrax vaccine because they believed that the vaccine was not effective in protecting their cattle against anthrax. Therefore, they were not willing to have their cattle vaccinated. This observation resonates with the postulation of the Health Belief Model. The Health Belief Model postulates that an individual is more likely to seek help or treatment when they see the efficacy of taking such action to reduce susceptibility or reduce the risk of disease (perceived benefits) (Burke 1950). The model has been applied to predict health-related behaviours such as vaccinations. Therefore, there is a need to sensitise farmers on the importance of cattle vaccinations. Vaccinating cattle during outbreaks should be avoided to address misconceptions concerning the efficacy of the vaccine.

Equally so, the community members did not seem to trust professional veterinary staff as they suspected them of practicing *Satanism*. The lack of trust by community members could be attributed to reduced access to information and lack of community engagement as expressed by community members. Some studies in other zoonotic diseases like Ebola have demonstrated similar trends (DingWall 2015). DingWall indicated that during the Ebola outbreak in West Africa, health professionals failed to collaborate with traditional healers, who were often more accessible and more trusted than government health systems. Public health communications were poorly designed and may well have promoted rather than reduced risk behaviour. Research evidence has shown that community involvement in control programmes should be fostered to enhance trust and cooperation (DingWall 2015). This finding confirms the importance of involving local communities in programmes to enhance cooperation and trust. DingWall (2015) therefore concluded that ‘community engagement is the one factor that underlies the success of all other control measures’. Therefore, the importance of community engagement cannot be overemphasised.

### Lay practices affecting anthrax transmission

The study also established that the communities were engaged in specific cultural practices that facilitated transmission of anthrax between animals. Firstly, the communal ownership of cattle made it difficult for herders to make timely decisions to vaccinate their animals. Other studies have indicated that most pastoral communities tend to have a communal ownership of cattle, which leads to difficulties in making decisions. People working in urban areas yet owning cattle back home leave their animals under the husbandry of caretakers on the floodplains (Munang’andu et al. 2012). When an animal dies in the absence of the owner, the caretaker would keep the head, hide or other suitable body parts as evidence that the animal was not sold but died of a disease when the owner returned. Such traditional practices predisposed both cattle and human beings to the risk of anthrax infection. Further, communities in the Western Province practiced the transhumance grazing system. The annual migration of cattle between the upland and the floodplains put anthropogenic pressures on the limited pastures and water points (Munang’andu et al. 2012). This can be attributed to the fact that during the dry season (May–October), most watering points tend to be dry, with only muddy water remaining in the oxbow lakes and lagoons apart from the main river channel. The grass is depleted from most of the floodplains except the dambos around the oxbow lakes and lagoons. Animals graze close to the ground and inhale the spores. It is also likely that they ingest spores by drinking muddy water that has been contaminated by spores persisting in the micro-environments around the oxbow lakes and lagoons that serve as the only sources of water. This situation could have facilitated the transmission of anthrax to animals. Although the farmers in the Western Province are not nomadic, the annual migration system promotes the transmission of diseases into new areas as is the case with the *Masai* of Tanzania (Mangesho et al. 2017).

Carcass disposal practices when an animal died also contributed to the transmission of animal anthrax. In the Western Province, it was common for community members to slaughter and eat meat from infected carcasses. Therefore, when cattle died, it was butchered and shared right from where it died. This practice promoted the dissemination of anthrax bacterium into the environment. Incineration was rarely practiced as professional staff did not commonly respond to calls for animal mortalities. Processing of animal products disseminated the spores even further to the immediate environment and beyond. A study conducted in Bangladesh also found that community members disposed of their carcasses in water bodies where humans and animals drank water (Hassan et al. 2015), leading to ingestion or inhalation of spores by other cattle. These findings underscore the importance of professional staff to supervise and educate community members on the safe disposal practices of the carcass. Tuchili et al. (1997) were able to isolate *B. anthracis* from preserved smoked dry meat in the Western Province, indicating that the processing and preservation methods employed do not inactivate the anthrax spores. Further studies carried out by Tuchili et al. (1997) showed the presence of anthrax spores from cattle hides that had been preserved and stored for a long time. This poses a significant public health threat, considering that large quantities of these products are sold to the general public outside the Western Province without any quality assurance tests and public health inspections, thereby increasing the risk of anthrax distribution from endemic to non-endemic urban areas every year.

Consistent with other studies (Chakraborty et al. 2012; Chirundu et al. 2009; Hassan et al. 2015), this study established that few farmers vaccinated their animals against anthrax. Participants in the focus group discussions cited various reasons for failure to have their animals vaccinated. The most significant ones were poverty, difficulties in accessing the vaccine, inadequate access to veterinary services and lack of cold chain facilities. Key informants mentioned logistical challenges such as lack of cold chain facilities, funding for anthrax control, reduced access to the vaccine and poor attitude of the farmers as some of the factors that perpetuated anthrax in the Western Province. These findings were consistent with other literature that postulates that anthrax affects poor communities with poor veterinary infrastructure and occurs among marginalised populations (Food and Agriculture Organisation of the United Nations 2003). Anthrax has also persisted in countries where public health infrastructure is weak. There is therefore need to improve public health infrastructure.

## Conclusion

The study established that anthrax infection in cattle was not only biologically determined but also culturally maintained. The study found that cattle owners were reluctant to have their cattle vaccinated because they believed that the vaccine made their animals sick. Furthermore, community members did not trust professional staff and their technical interventions. Popular cultural practices that involved exchange of animals between herds and uncontrolled animal movements contributed to the transmission of anthrax between cattle.

We therefore argue that current technical approaches to control anthrax must be backed by the social, and cultural, framework. Also, there must be strategic annual vaccinations of cattle coupled with improved public health awareness campaigns aimed at promoting active participation by the general public in the control of the disease. Lastly, there is a need to improve veterinary extension services and restore veterinary infrastructure to control animal movements in the province.

## References

[CIT0001] Burke E, 1950, *The health belief model*, pp.1–3, viewed 09 October 2016 from https://www.iccwa.org.au/useruploads/files/soyf/2013_resources_videos/the_health_belief_model.pdfevan_burke.pdf

[CIT0002] Central Statistical Office Zambia, 2014, *Zambia demographic and health survey, 2013–2014*, p. 518 Lusaka: Government Printers.

[CIT0003] Chanda K.N., Mulubwe B. & Mwale F, 2017, ‘Outbreak of anthrax among humans and cattle in Western province of Zambia, November 2016 to January 2017’, *Health Press Zambia Bull*, 1(1), 50–55.

[CIT0004] Chakraborty A., Khan S.U., Hasnat M.A., Parveen S., Islam S.M., Mikolon A., Kumar R., Ahmed C.B., Ara K.,Haider N., Zaki S.R., Hoffmaster A.R, Rahman M., Luby S.P. & Hossain M.J, 2012, ‘Anthrax Outbreaks in Bangladesh, 2009–2010’, 86(4), pp. 703–710. 10.4269/ajtmh.2012.11-0234 PMC3403762 22492157

[CIT0005] Chavwanga V, 2013, *The department of veterinary services and control of contagious diseases*, University of Zambia. Lusaka: University of Zambia Press.

[CIT0006] Chirundu D., Chihanga S., Chimusoro A., Chirenda J. & Apollo M.T, 2009, ‘The central african’, *The Central African Journal of Medicine* 55, 50–54. 21977844 10.4314/cajm.v55i9-12.63640

[CIT0007] DingWall R, 2015, ‘Ebola – WHO (Still) don’t get it: Social science saves lives’, *Social Science Space*, viewed 05 September 2016 from http://www.socialsciencespace.com/2015/02/ebola-who-still-dont-get-it-social-science-saves-lives/

[CIT0008] Food and Agriculture Organization of the United Nations, 2003, ‘EMPRES transboundary animal diseases bulletin’, *EMPRES Transboundary Animal Diseases Bulletin*, 24, 2–7.

[CIT0009] Hassan J., Ahsan M., Rahman B., Chowdhury Z.H., Parvej S. & Nazir K.H.M.N.H, 2015, ‘Factors associated with repeated outbreak of anthrax in Bangladesh: Qualitative and quantitative study’, *Journal of Advanced Veterinary and Animal Research*, 2, 158–164. https://doi.org/10.5455/javar.2015.b72

[CIT0010] Hugh-Jones M.E. & Hussaini S.N, 1975, ‘Anthrax in England and Wales, 1963–1972’, *Veterinary Record*, 97, 256–261. https://doi.org/10.1136/vr.97.14.256 1179615 10.1136/vr.97.14.256

[CIT0011] IUCN, 2003, ‘Barotse Floodplain, Zambia: Local economic dependence on wetland resources’, in *Case studies in wetland valuation*, viewed 13 November 2016 from http://www.cbd.int/doc/case-studies/inc/cs-inc-iucn-12-en.pdf

[CIT0012] Kamboyi H.K, 2015, *No title risk mapping and eco-anthropological assessment of anthrax in the upper Zambezi*, University of Zambia, Lusaka.

[CIT0013] Mangesho P.E., Neselle M.O., Karimuribo E.D., Mlangwa J.E., Queenan K., Mboera L.E. et al., 2017, ‘Exploring local knowledge and perceptions on zoonoses among pastoralists in northern and eastern Tanzania’, *PLoS Neglected Tropical Diseases*, 11(2), 1–22. https://doi.org/10.1371/journal.pntd.0005345 10.1371/journal.pntd.0005345 PMC5325590 28146556

[CIT0014] Mumba C., Häsler B., Muma J.B., Munyeme M., Sitali D.C., Skjerve E. et al., 2018, ‘Practices of traditional beef farmers in their production and marketing of cattle in Zambia’, *Tropical Animal Health and Production*, 50(1), 49–62. https://doi.org/10.1007/s11250-017-1399-0 28948428 10.1007/s11250-017-1399-0

[CIT0015] Munang’andu H.M., Banda F., Siamudaala V.M., Munyeme M., Kasanga C.J. & Hamududu B, 2012, ‘The effect of seasonal variation on anthrax epidemiology in the upper Zambezi floodplain of western Zambia’, *Journal of Veterinary Science*, (3), 293–298. https://doi.org/10.4142/jvs.2012.13.3.293 23000586 10.4142/jvs.2012.13.3.293 PMC3467405

[CIT0016] Muuka G., Songolo N., Kabilika S., Hang’ombe B.M., Nalubamba K.S. & Muma J.B, 2012, ‘Challenges of controlling contagious bovine pleuropneumonia in sub-Saharan Africa: A Zambian perspective’, *Tropical Animal Health and Production*, 45(1), 9–15. https://doi.org/10.1007/s11250-012-0235-9 22843213 10.1007/s11250-012-0235-9

[CIT0017] Mwambi P.E.M., Mufunda J., Mwaba P., Kasese-Chanda N., Mumba C.M., Kalumbi T. et al., 2017, ‘Cutaneous anthrax outbreak in Chama district, Muchinga province, Zambia’, *Health Press Zambia*, 1(1), 38–49.

[CIT0018] National Livestock Epidemiology and Information Centre, 2015, *Department of Veterinary Services Annual Report*, Lusaka: Government Printers.

[CIT0019] New M., Lister D., Hulme M., & Makin I, 2002, ‘A high-resolution data set of surface climate over global land areas’, *Climate Research*, 21, 1–25.

[CIT0020] Office of the Auditor-General, 2015, *Report of the Auditor General on the Management and Control of Livestock Diseases*, Lusaka: Government Printers.

[CIT0021] Siamudaala V.M., Bwalya J.M., Munang’andu H.M., Sinyangwe P.G., Banda F. & Mweene A.S, 2006, ‘Ecology and epidemiology of anthrax in cattle and humans in Zambia’, *The Japanese Journal of Veterinary Research*, 54(1), 15–23, viewed 17 September 2014 from http://www.ncbi.nlm.nih.gov/pubmed/16786974 16786974

[CIT0022] Sitali D.C., Mumba C., Skjerve E., Mweemba O., Kabonesa C. & Mwinyi M.O, 2017, ‘Awareness and attitudes towards anthrax and meat consumption practices among affected communities in Zambia: A mixed methods approach’, *PLoS Neglected Tropical Diseases*, 11(5), e0005580 https://doi.org/10.1371/journal.pntd.0005580 28498841 10.1371/journal.pntd.0005580 PMC5443538

[CIT0023] Siziya S, 2017, ‘Anthrax outbreaks and epidemics in Zambia, 1990–2011: A review’, *Health Press Zambia Bull*, 1(1), 21–27.

[CIT0024] Sumbwa N, 2000, ‘Traditionalism, democracy and political participation: The case of western province’, *African Study Monographs*, 21, 105–146.

[CIT0025] Tuchili L.M., Pandey G.S., Sinyangwe P.G. & Kaji T, 1993, ‘Anthrax in cattle, wildlife, and humans in Zambia’, *Veterinary Record*, 132, 487 https://doi.org/10.1136/vr.132.19.487-a 10.1136/vr.132.19.487-a 8506601

[CIT0026] Turpie J.B, 2004, ‘The value of floodplain fisheries in the Zambezi river basin’, *International Workshop on the Fisheries of the Zambezi Basin*, World Fish Center, Malawi, May 31, 2004.

[CIT0027] Welcomme R.L, 1975, ‘The fisheries ecology of African floodplains L’ecologie des pêches dans les plaines inondables africaines’, Food and Agriculture Organization (FAO) of the United Nations, CITA Tech Pap, vol. 3, p. 51, viewed 02 January 2018 from http://www.fao.org/docrep/005/f9051e/F9051E00.htm#TOC

[CIT0028] World Anthrax Data Site, 2007, *A service for the World Health Organization Collaboration Center for Remote Sensing and Geophysical Information systems for Public Health*, Anxieties over anthrax, viewed 16 February 2016 from http://www.vetmed.1su.edu/whocc/mp_world.htm

